# Clinical Trial Protocol for BEACH: A Phase 2a Study of MW189 in Patients with Acute Nontraumatic Intracerebral Hemorrhage

**DOI:** 10.1007/s12028-023-01867-2

**Published:** 2023-11-02

**Authors:** Radhika Avadhani, Wendy C. Ziai, Richard E. Thompson, W. Andrew Mould, Karen Lane, Angeline Nanni, Michael Iacobelli, Matthew F. Sharrock, Lauren H. Sansing, Linda J. Van Eldik, Daniel F. Hanley, on behalf of the BEACH trial consortium, Aaron Lord, Elizabeth Liptrap, Mario Zuccarello, Kevin Hatton, Tarun Girotra, Tiffany Chang, Justin Mascitelli, Jessica Magid-Bernstein, Marc Babi

**Affiliations:** 1 BIOS Clinical Trials Coordinating Center, Johns Hopkins School of Medicine, 750 East Pratt Street, 16th Floor, Baltimore, MD 21202, USA; 2 Division of Neurocritical Care, Department of Anesthesiology and Critical Care Medicine, Johns Hopkins School of Medicine, Baltimore, MD USA; 3 Department of Biostatistics, Johns Hopkins Bloomberg School of Public Health, Baltimore, MD USA; 4 Division of Neurocritical Care, Department of Neurology, University of North Carolina at Chapel Hill, Chapel Hill, NC USA; 5 Department of Neurology, Yale University School of Medicine, New Haven, CT USA; 6 Sanders-Brown Center on Aging and Department of Neuroscience, University of Kentucky, Lexington, KY USA

**Keywords:** Intracerebral hemorrhage, Clinical trial, MW01-6-189WH, MW189, Radiographic perihematomal edema, Neuroinflammation, Cerebral edema

## Abstract

Patients with acute spontaneous intracerebral hemorrhage (ICH) develop secondary neuroinflammation and cerebral edema that can further damage the brain and lead to increased risk of neurologic complications. Preclinical studies in animal models of acute brain injury have shown that a novel small-molecule drug candidate, MW01-6-189WH (MW189), decreases neuroinflammation and cerebral edema and improves functional outcomes. MW189 was also safe and well tolerated in phase 1 studies in healthy adults. The proof-of-concept phase 2a Biomarker and Edema Attenuation in IntraCerebral Hemorrhage (BEACH) clinical trial is a first-in-patient, multicenter, randomized, double-blind, placebo-controlled trial. It is designed to determine the safety and tolerability of MW189 in patients with acute ICH, identify trends in potential mitigation of neuroinflammation and cerebral edema, and assess effects on functional outcomes. A total of 120 participants with nontraumatic ICH will be randomly assigned 1:1 to receive intravenous MW189 (0.25 mg/kg) or placebo (saline) within 24 h of symptom onset and every 12 h for up to 5 days or until hospital discharge. The 120-participant sample size (60 per group) will allow testing of the null hypothesis of noninferiority with a tolerance limit of 12% and assuming a “worst-case” safety assumption of 10% rate of death in each arm with 10% significance and 80% power. The primary outcome is all-cause mortality at 7 days post randomization between treatment arms. Secondary end points include all-cause mortality at 30 days, perihematomal edema volume after symptom onset, adverse events, vital signs, pharmacokinetics of MW189, and inflammatory cytokine concentrations in plasma (and cerebrospinal fluid if available). Other exploratory end points are functional outcomes collected on days 30, 90, and 180. BEACH will provide important information about the utility of targeting neuroinflammation in ICH and will inform the design of future larger trials of acute central nervous system injury.

## Introduction

Nontraumatic spontaneous intracerebral hemorrhage (ICH) is an important public health problem, leading to high mortality rates and morbidity in adults, resulting from rupture of a cerebral blood vessel due to arteriolosclerosis or cerebral amyloid angiopathy [1, 2]. Primary ICH is the second most common subtype of stroke, with an annual incidence of 10–30 cases per 100,000 people, accounting for 10–15% of approximately 15 million strokes worldwide every year [3]. In the United States, the overall incidence of ICH is approximately 40,000 to 67,000 per year [4]. Of all stroke subtypes, ICH is associated with the highest mortality rate (~ 25–50%) and morbidity (~ 80% functional disability) at 1 year [4, 5]. Incidence increases significantly among individuals > 75 years old, a population in which mortality has not declined compared to younger patients [6–8], possibly because of increased prevalence of vascular comorbidities, cerebral amyloid angiopathy, and hypertension. Thus, aging of the world’s population makes ICH a major health challenge, requiring more effective therapeutics to improve survival rate, functional outcomes, and quality of life [9–12].

Brain injury after ICH includes both the primary injury caused by the hemorrhage and hematoma expansion and secondary injury caused by the brain’s response to the hematoma, primarily perihematomal edema (PHE) and inflammation. In ICH, a robust increase in cytokine levels in the brain occurs in the first few days after injury, providing a well-defined therapeutic window for targeting the dysregulated neuroinflammatory response and a health care delivery time window that allows feasible intervention after patient presentation to the hospital. Preclinical and clinical research strongly suggests this acute proinflammatory cytokine surge is a key contributor to cerebral edema, long-term neurological damage, and cognitive deficits following acute brain injuries [6, 13, 14]. The mechanistic linkage of the acute cytokine surge to progression of ICH injury, plus the attractive therapeutic time window of hours to days post injury, provides a rational therapeutic target and scientific premise for intervention in the acute care setting.

MW189 is a novel small-molecule investigational drug candidate developed as a selective suppressor of disease and injury-induced proinflammatory cytokine overproduction associated with destructive neuroinflammation/synaptic dysfunction cycles [15, 16]. MW189 restores the activated glial signaling pathways back toward homeostasis. MW189 in experimental models demonstrates efficacy at low doses and is a strong inhibitor of glial production of proinflammatory cytokines and chemokines relevant to neuroinflammatory disorders, such as ICH and traumatic brain injury [17]. Therefore, we designed a first-in-patient exploratory trial with the aim to determine safety and tolerability of MW189 in participants with acute spontaneous ICH. The Biomarker and Edema Attenuation in IntraCerebral Hemorrhage (BEACH) study also includes exploratory outcome measures that will provide initial evidence for the effect of MW189 on PHE and on clinical outcomes up to 180 days. The study will evaluate pharmacokinetics and pharmacodynamics of MW189 in plasma and cerebrospinal fluid (in participants with an external ventricular drain), including inflammatory and neurologic dysfunction protein profiles. Data from this study will inform a future phase 2b/3 trial.

In a first-in-human phase 1a single ascending dose study, MW189 was administered as a single intravenous (IV) dose at 0.025, 0.05, 0.1, or 0.25 mg/kg (15- to 20-min infusion) to 24 healthy adult participants (female and male) and was well tolerated [18]. No serious adverse events (AEs) or discontinuations due to AEs occurred. Reported AEs were mild and resolved without intervention. Incidence of treatment-related AEs did not rise with higher MW189 doses. No AEs tied to laboratory test results, vital signs, electrocardiography, or infusions were noted. No treatment or dose trends were observed. MW189’s pharmacokinetic profile was consistent across doses, with linear kinetics and no significant accumulation. In a pilot phase 1a study of endotoxin-induced changes in plasma cytokine levels in healthy male volunteers, a single IV dose (0.25 mg/kg) of MW189 resulted in lower levels of the proinflammatory cytokine tumor necrosis factor-α and increased levels of the antiinflammatory cytokine interleukin-10 compared with placebo treatment [18].

A phase 1b multiple ascending dose study [18] of eight healthy participants (male/female) per dose cohort randomized to MW189 (0.075, 0.15, 0.25, or 0.30 mg/kg IV twice a day [or matched saline placebo control]) for 5 days consecutively, given 12 h apart, reported that MW189 was safe and well tolerated. The MW189 group showed milder to moderate infusion-related AEs (e.g., extremity pain, infusion site pain), with minimal laboratory test result, vital signs, and electrocardiogram changes. No significant trends or postdose findings were noted. MW189 displayed linear kinetics, with dose-related increases in maximum drug concentration (*C*_max_) and area under the curve over 12-h dosing across cohorts. Steady state was achieved after the third dose in lower groups. These findings support MW189’s safety and pharmacokinetic and pharmacodynamic effects for acute brain injury development.

In this BEACH phase 2a trial, participants will be recruited from October 2022 through December 2025, with anticipated data collection ending in June 2026. This study is planned to last 5 years: 9 months for preenrollment activities (institutional review board [IRB] approval, site initiation, and activation), followed by enrollment of 120 participants, each with 180 days of follow-up, and 6 months to analyze outcomes and develop the study report.

## Methods

### Study Design

The BEACH trial (ClinicalTrials.gov identifier: NCT05020535) is a multicenter, randomized, double-blind, placebo-controlled phase 2a clinical trial conducted at 10 US clinical sites (supplementary material). All sites are comprehensive stroke centers and were selected from Clinical and Translational Science Awards Program sites. BEACH is directed by the University of Kentucky and the Johns Hopkins University BIOS Clinical Trials Coordinating Center. The Johns Hopkins Medicine IRB, serving as the single IRB, approved the study design. The trial will be conducted in accordance with the principles of the Declaration of Helsinki, the Good Clinical Practice guidelines of the International Council for Harmonisation, and all applicable regulatory requirements.

### Patient Population

A total of 120 participants (60 per group) with acute spontaneous nontraumatic ICH are eligible for this study. The inclusion and exclusion criteria are detailed in Table 1. Written informed consent will be obtained from all participants or their legally authorized representatives.

**Table 1:** Inclusion and exclusion criteria

Inclusion criteria: a patient will be eligible for inclusion in the study only if all of the following criteria are met:	
Confirmed diagnosis of spontaneous nontraumatic ICH	
10 mL ≤ ICH ≤ 60 mL (confirmed via diagnostic and stability CT scans using volumetric assessment)	
Participants receiving anticoagulants are eligible upon reversal and stability within 24 h after onset of ICH symptoms	
Age ≥ 18 years	
Able to receive first dose of test article (drug or placebo) ≤ 24 h after onset of ICH symptoms	
NIH Stroke Scale score ≥ 2 at randomization or Glasgow Coma Scale score ≥ 5 at randomization	
Controlled blood pressure (systolic blood pressure ≤ 160 mm Hg) at randomization	
Premorbid modified Rankin Scale score of 0–2	
Has adequate venous access	
No planned surgical intervention except external ventricular drain	
Written informed consent from the patient or legally authorized representative	
Exclusion criteria: a patient will not be eligible for inclusion in the study if any of the following criteria are met:	
Unstable hematoma defined as > 6-mL increase as compared to previous CT volume taken at least 6 h apart within 24 h after onset of ICH symptoms	
Anticipated neurosurgical evacuation by open surgery or minimally invasive surgery with or without alteplase (external ventricular drain allowed)	
Uncontrolled temperature > 38.5 °C at enrollment	
Signs of intracranial infection or emergence of a systemic infection	
Is pregnant or lactating	
Signs of liver and kidney chronic disease (i.e., creatinine > 2 mg/dL, bilirubin ≥ 3 mg/dL, receiving dialysis)	
Nonreversible bleeding diathesis	
Used any chronic immunosuppressants or chronic antiinflammatory drugs (excluding low-dose aspirin) by any route of administration within the past 7 days	
Anticipated withdrawal of life-sustaining therapies within the first week after admission	
In the opinion of the investigator, patient has any contraindication to the planned study assessments	
In the opinion of the investigator, patient has a condition that could interfere with the proposed treatment or that could unacceptably increase the individual’s risk by participating in the study	
Thrombolytic-associated ICH or hemorrhagic conversion of an ischemic stroke, along with other causes of secondary ICH	
Concomitant enrollment in another acute interventional study	

CT, computed tomography, ICH, intracerebral hemorrhage, NIH, National Institutes of Health

### Streamlined Patient Screening

Traditionally, clinical sites have used linear and semiquantitative estimates of ICH and intraventricular hemorrhage (IVH) volume, such as ABC/2 [19], to assess eligibility for clinical trials. Errors in volume measurements occur without training and increase with large or irregularly shaped ICHs [20, 21]. More time-intensive methods, such as computed-tomography-based planimetry (CTP), demonstrate good accuracy within 5 mL or 20% of CTP volume. Recent research has demonstrated that novel concepts, such as the use of brain atlases, heuristic image quantitation, and machine learning, could provide increased diagnostic precision in volumetric assessments for patients with ICH and IVH. These methods yield significant time savings compared to traditional ABC/2 and CTP methods, potentially increasing enrollment rates for clinical studies of patients prescreened for ICH or IVH [21–23]. The BEACH trial uses the Viz RECRUIT platform, which uses artificial intelligence to expedite the screening process for ICH trials through automated scanning of patient computed tomography (CT) images in real time, allowing for precise volumetric assessments for suspected patients with ICH, identification of potential participants, and immediate notification of potential patients to research teams [24]. Currently, there is no US Food and Drug Administration (FDA) clearance for the ICH volume measurement. The FDA-cleared ICH commercial product is indicated for ICH detection and notification. Automatic segmentation and volumetric calculation are for investigational use only and are included in the separate research/clinical trial platform called RECRUIT [25].

### Randomization and Blinding

Randomization will be performed by a computer-generated randomization schedule that will assign participants 1:1 to either MW189 or placebo. The allocation sequence will be created and stored in a protected electronic data capture system. All providers and nurses participating in the treatment plan will be excluded from data and outcome analyses. The observers who assess the clinical outcomes will be blinded to the randomization results. A safety review will be performed in a blinded manner unless the data warrants unblinding because of the nature of any safety concerns. When unblinding is required, requests will be directly forwarded to the study’s unblinded statistician by the site investigator. To further maintain blinding during study drug administration, the IV equipment will be covered. Once the study drug is diluted, it must be stored at ambient temperature and protected from light until administration.

### Study Intervention

**Fig. 1:**
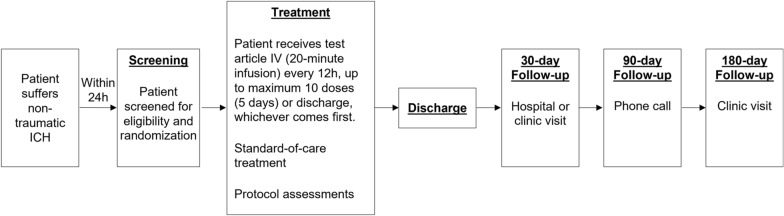
Schematic of study design. ICH, intracerebral hemorrhage; IV, intravenously

The intervention will start within 24 h of symptom onset in the intensive care unit or hospital ward (Fig. 1). Participants will be monitored daily throughout the treatment phase (randomization through day 5 or discharge if sooner) and will receive guideline-based usual care treatment for the duration of the study. Usual care treatment will follow American Heart Association guidelines for the management of ICH [26]. Participants will receive either MW189 (0.25 mg/kg) or saline administered IV over approximately 20 min every 12 ± 2 h for up to 5 days (10 doses) or until discharge, whichever comes first. University of Iowa Pharmaceuticals, an FDA-registered pharmaceutical contract manufacturing organization, prepared the MW189 drug product. It is a sterile concentrated solution in 0.9% sodium chloride at 2.5 mg/mL with a pH of roughly 2.4 that is diluted in saline prior to IV administration. The concentration of the diluted solution must not exceed 0.4 mg/mL, which will confirm that the minimum solution pH is 3.0. At a dose of 0.25 mg/kg and a diluted solution concentration of 0.4 mg/mL, the volume for a 50- to 120-kg adult will be 31–75 mL over a 20-min infusion time. Participants randomized to placebo will receive 0.9% sodium chloride, USP (US Pharmacopeia), at a volume equal to that of the active treatment as calculated based on the participant’s weight.

### Follow-Up Time

At each study site, trained research coordinators blinded to treatment assignments will perform follow-up visits and calls. Follow-up clinic visits will be done at 30 ± 3 days and 180 ± 14 days after drug last dose. Follow-up phone calls will be done at 90 ± 3 days after drug last dose.

### Study Outcomes

The primary study outcome is the proportion of deaths from all causes during the first 7 days post randomization. Secondary outcomes include all-cause mortality at 30 days, hematoma expansion and recurrent ICH, brain infection, standard pharmacokinetic parameters (time to maximum plasma concentration [*T*_max_], *C*_max_, area under the curve, half-life [*t*_1/2_], clearance [CL], volume of distribution during terminal phase [*V*_z_], apparent terminal elimination rate constant [λ_z_]) determined at different times after MW189 administration, radiographic measures of edema during treatment and at the end of treatment for days 1–5, and measures of plasma and cerebrospinal fluid inflammatory and neuronal injury biomarkers during treatment and at the end of treatment for days 1–5. We will use a semiautomated threshold approach (range 44–100 Hounsfield units) to determine hematoma volume. Significant hematoma expansion will be defined as an increase between baseline and any follow-up CT scan of > 6 mL or > 33%. IVH volume will not be included in hematoma expansion. Recurrent ICH refers to the emergence of a new ICH following an initial episode of spontaneous ICH. It is subsequent spontaneous bleeding in the brain distinct from the initial event, typically occurring after a time frame of around 30 days or more. This is different from ICH expansion, which involves the enlargement of the initial bleed without the occurrence of a completely new bleeding event. Brain infection refers to infection present within the brain tissue and is evaluated based on the clinical symptoms, laboratory test results, and imaging findings. Radiographic measures of edema refer to the quantitative assessment of PHE and will be analyzed via both CT and magnetic resonance imaging (MRI) scans using semiautomated volumetrics with OsiriX software to compare the volume of edema between the two treatment groups. The primary imaging outcomes are absolute PHE and relative PHE and edema extension distance measured on brain CT scans performed at admission and at day 5 of the infusion. PHE measurements on MRI will be performed on the subset with MRI at baseline and at the end of the study. Group differences in edema volumes will be compared.

Exploratory end points include clinical and functional outcomes collected at days 30, 90, and 180 (assessed using dichotomized modified Rankin Scale scores of 0–3 vs. 4–6, the National Institutes of Health Stroke Scale, the Montreal Cognitive Assessment, the Barthel Index, the Stroke Impact Scale, and the Center for Epidemiologic Studies-Depression scale) and participant status at 180 days from ICH, including serious AEs, AEs of special interest, hemorrhage expansion and recurrent ICH, infections, and mortality.

### Data and Safety Monitoring

We have established an independent Data and Safety Monitoring Board (DSMB) to ensure the safety of enrolled participants. The DSMB review will occur every 6 months or independently after enrollment of 12, 30, 60, and 120 participants. Safety events that reach or exceed the prespecified threshold will trigger “suspend recruitment and review” by the DSMB to further investigate any presumed cause and impact of any events. Termination or suspension circumstances include determining unexpected or unacceptable risks, demonstrating significant efficacy, inadequate compliance to the protocol, incomplete or inconclusive data, meeting the primary end point, or establishing futility. The trial also includes monitoring events of special interest, such as phlebitis and infection. If 75% of all participants experience one or more of these events, the DSMB will be notified for review. This assessment will be conducted after the enrollment of the first ten participants. All clinical trial data, including all AEs, will be recorded on the case report form through day 5 or discharge, whichever occurs first.

### Sample Size Determination

The primary outcome of the study is to measure the proportion of participant deaths from any cause within the first 7 days after randomization. We anticipate that the mitigation of inflammation and edema will lead to fewer deaths in the MW189 arm during the acute phase. However, with an abundance of caution, we assume the placebo arm will have a similar number of deaths as compared to the MW189 arm. Therefore, we conducted a noninferiority power analysis using previously collected data from the Minimally Invasive Surgery Plus Alteplase for Intracerebral Hemorrhage Evacuation (MISTIE) III [27] and Antihypertensive Treatment of Acute Cerebral Hemorrhage II (ATACH-2) trials [28] to approximate the safety events that will be seen in the trial.

Considering a power of 80% and a one-sided 90% confidence interval, to test the null hypothesis of noninferiority with a tolerance limit of 12% and assuming a “worst-case” safety assumption of 10% rate of death in each arm, the sample size per group was estimated to be 57. We assume a low overall withdrawal rate in the first 7 days, and therefore 120 participants (60 per group) are required to evaluate the safety of MW189. This number also meets the estimated sample size for an 80% power and two-sided *α* to detect various assumed differences in PHE volume and maximum observed effect (*E*_max_) values in the interleukin-10 plasma cytokine response between treatment arms.

### Statistical Analyses

The intention-to-treat analysis set (all randomized participants) will be used for efficacy analysis. All participants who receive any study treatment will be included in the safety analyses. Continuous variables of normal distribution will be expressed as mean ± standard deviation, whereas continuous variables of nonnormal distribution will be expressed as median and interquartile range. Categorical variables will be represented as frequencies and percentages. We anticipate no missing data for the primary outcome, as all-cause mortality within 7 days of treatment initiation is verifiable.

The primary outcome will involve an estimate of the difference in the proportion of all-cause mortality within 7 days of treatment initiation using a 90% one-sided confidence interval on the upper bound of this difference. The null hypothesis will be rejected if this confidence interval is less than 10%. In addition, we will also estimate the treatment difference in the proportion of 7-day all-cause mortality using a multivariable logistic regression model. This model will consider age, sex, race and ethnicity, ICH volume at stability, Glasgow Coma Scale (GCS) score, presence of IVH, withdrawal of life-sustaining therapy, and ICH location (deep versus lobar). This trial encompasses infratentorial ICH, which comprises brainstem ICH classified as deep location and cerebellar ICH classified as lobar location. Age, sex, and race and ethnicity data are collected at the screening visit. The GCS score is measured at baseline. Presence of IVH and ICH location are determined based on the diagnostic CT scan. The ICH volume assessed on the stability CT scan determines the need for additional imaging to establish hematoma at 6-h intervals until the end of the enrollment period. Withdrawal of life-sustaining therapy information is collected at any time during the study period. The schedule of activities is detailed in Table 2.

**Table 2:** Schedule of activities

Activities	Screening/enrollment first dose	Treatment				Follow-up		
Day	1	2	3	4	5	30 (clinic)	90 (phone)	180 (clinic)
Time frame	First dose within 24 h of ICH onset							
Pre-randomization								
Diagnostic CT	X							
CTA/MRI to rule out underlying lesion	X							
Inclusion/exclusion criteria review	X							
Consent by participant or legally authorized representative	X							
MRI to rule out underlying lesion	X							
Stability CT	X							
Pregnancy test	X							
Demographics	X							
Medical history and admission information	X							
Concomitant medications review	X							
At randomization								
Vital signs	X							
Glasgow Coma Scale	X							
Baseline (premorbid) modified Rankin Scale (caregiver interview)	X							
Post randomization								
Research MRI					X			
Usual care clinical CT		X	X	X				
Research CT					X			
Therapy intensity level	X	X	X	X	X			
National Institutes of Health Stroke Scale	X	X	X	X	X	X		X
Glasgow Outcome Scale-Extended						X	X	X
Glasgow Coma Scale	X	X	X	X	X	X		X
Montreal Cognitive Assessment					X	X		X
Stroke Impact Scale-16					X	X		X
Barthel Index	X				X	X	X	X
Modified Rankin Scale						X	X	X
EQ-5D						X	X	X
Center for Epidemiological Studies–Depression						X	X	X
Concomitant medications review		X	X	X	X	X	X	X
Plasma biomarkers	X		X		X			
Cerebrospinal fluid biomarkers	X		X		X			
Pharmacokinetic blood samples	X	X	X		X			
Dosing	X	X	X	X	X			
Adverse events assessment	X	X	X	X	X	SAE/MEOI	SAE	SAE

CT, computed tomography; CTA, computed tomography angiography; ICH, intracerebral hemorrhage; MEOI, medical event of interest; MRI, magnetic resonance imaging; SAE, serious adverse events

Secondary and exploratory safety outcomes will be analyzed using appropriate linear or logistic regression models and will account for treatment group, age, GCS score, presence of IVH, ICH volumes, the timing of drug administration, and hematoma location. No imputation of missing safety end points will be used. Instead, sensitivity analyses will be performed, whereby participants with missing secondary safety data will be assumed to have had a safety event and then not to have had a safety event. The analyses will account for the variability in dosing and treatment duration, which can range from 2 to 10 doses depending on the date of discharge. This variability will be addressed through various approaches, such as categorizing participants into dose groups, treating the number of doses as a continuous variable, and calculating area under the curve that considers the dose and duration of the doses given. The point estimate of the proportion of total safety events and corresponding two-sided 95% confidence intervals will be calculated under both extreme scenarios to provide estimates of uncertainty in the presence of these missing data. SAS, STATA 17, and/or R software will be used for statistical analysis. Statistical significance will be set at *p* < 0.05.

## Discussion

The primary objective of the BEACH trial is to assess if five consecutive days of MW189 treatment after spontaneous ICH is safe and does not increase early mortality. Prior nonclinical studies demonstrated that this novel investigational small-molecule drug candidate reduces a set of central nervous system inflammatory responses after brain injury by suppressing the production of excessive glial proinflammatory mediators. In an ICH mouse model, 5-day treatment with low doses of MW189 (1 mg/kg) significantly decreased cerebral edema post injury and reduced vestibulomotor deficits [17]. In preclinical safety, pharmacology, and toxicology studies, an IV formulation of MW189 was developed based on the standard route of administration in acute brain injury trials. Phase 1 clinical studies performed with this IV drug product demonstrated good tolerance and no treatment-related or treatment-emergent AEs, especially in vital organ systems. Importantly, when considering the anticipated pharmacologic effect of MW189, no alterations were observed in standard immunological parameters, including organ weights, hematology/blood cell counts, or microscopic and macroscopic evaluation of lymphoid and related tissues.

We added several points to the justification of the primary end point, time window, duration of treatment, and volume range of qualifying ICH. The selection of all-cause 7-day mortality as the primary outcome, the time window and duration of treatment with MW189, and the volume range of qualifying ICH (10–60 mL) are carefully justified based on clinical relevance and considerations and available preclinical and clinical evidence. First, the choice of all-cause 7-day mortality as the primary outcome in this trial is justified by its clinical significance and relevance in assessing the immediate impact of the intervention on early mortality rates. This end point provides valuable insights into the potential benefits of MW189 as a therapeutic intervention during the critical initial phase of acute spontaneous ICH. Second, regarding the time window and duration of treatment, preclinical studies and initial clinical data provided evidence of the drug’s therapeutic effects within a specific treatment duration [6, 13, 14]. By administering MW189 within 24 h of symptom onset and continuing treatment every 12 h for up to 5 days or until hospital discharge, we aim to maximize the drug’s potential neuroinflammation-reducing and cerebral-edema-attenuating effects. Lastly, the volume range of qualifying ICH between 10 and 60 mL was chosen because it reflects a clinically relevant and diverse range that encompasses a substantial proportion of spontaneous ICH cases. This allows for a comprehensive evaluation of MW189’s efficacy and safety across a diverse patient population with varying degrees of ICH severity, ensuring the generalizability of study findings.

Enrollment began in October 2022, and the estimated recruitment completion date is December 2025. Nine participants have been enrolled for the study as of August 2023. Recruitment will continue until the total sample size is achieved. There are no planned interim analyses.

MW189 holds promise for treating neuroinflammation in acute spontaneous ICH. BEACH, the first-in-patient, multicenter, placebo-controlled phase 2a trial assessing safety of MW189 in participants with ICH, will provide critical information about designing a larger phase 2b/3 trial to investigate efficacy of MW189. In addition, our secondary and exploratory analyses may identify mechanisms and biological responses to this new potential therapy targeted toward neuroinflammation and cellular brain injury.

## Clinical Trial Registration

ClinicalTrials.gov identifier NCT05020535.
